# Activity-induced reactivity of astrocytes impairs cognition

**DOI:** 10.1371/journal.pbio.3002712

**Published:** 2024-07-12

**Authors:** Kirsten Bohmbach, Christian Henneberger

**Affiliations:** 1 Institute of Cellular Neurosciences, Medical Faculty, University of Bonn, Bonn, Germany; 2 German Center for Neurodegenerative Diseases (DZNE), Bonn, Germany

## Abstract

Astrocytes can modulate neuronal activity and also acquire a reactive phenotype that correlates with cognitive impairments in brain diseases. A study in PLOS Biology shows that prolonged activation of astrocytes can trigger both cognitive impairments and a reactive astrocyte phenotype.

## Teaser

Glial cells such as astrocytes can modulate neuronal signaling. Astrocytes can also acquire a reactive phenotype that correlates with cognitive impairments in brain diseases. A study in *PLOS Biology* shows that prolonged activation of astrocytes can trigger both cognitive impairments and a reactive astrocyte phenotype.

In brain tissue, neurons with their dendrites and axons and glial cells like astrocytes and their processes are tightly interwoven. This enables astrocytes to support neuronal function by local clearance of neurotransmitter, by metabolite supply and by contributing to ion homeostasis. It also allows both cell types to communicate bidirectionally. Astrocytes, for example, can sense neuronal activity and modify synaptic information exchange between neurons by many different cellular mechanisms (for review [1,2]). For this reason, they can also modulate cognitive processes such as memory formation, retrieval, and extinction. However, it remains difficult to clearly associate a particular cellular mechanism with a specific aspect of memory (for review [3]). This is at least partly because it often remains unclear which cellular mechanisms are engaged by the experimental manipulation of astrocytes that eventually leads to a change of memory function. Optogenetic and chemogenetic manipulations of astrocytes have been particularly popular and effective in demonstrating a role of astrocytes for complex behaviors and memory (for review [3]) and both have been shown to augment memory [4,5].

In a new study, Kim and colleagues [6] now demonstrate that repeated and prolonged optogenetic stimulation of hippocampal astrocytes over a time period of days impairs the performance of mice in tests for spatial memory, working memory, and passive fear avoidance. Similarly, they found that repeated chemogenetic stimulation of astrocytes also reduced the performance in a passive fear avoidance test. Notably, cognitive impairment was only detected when the duration of individual optogenetic stimulations was relatively long and it was repeated for at least 3 days, or a strong chemogenetic stimulation was delivered repeatedly. In contrast, shorter and/or weaker stimulations were ineffective. In light of previous studies reporting both improvement and/or impairment of animals in memory tests by chemogenetic and optogenetic astrocyte stimulation [3–5], this emphasizes the need for careful consideration of the stimulation protocols (see also comparison by Kim and colleagues [6]), in addition to the attention to cell type specificity and potential side effects (e.g., virus injections, surgery, photostimulation) as performed by Kim and colleagues. To understand the variability of outcomes of astrocyte stimulation/manipulation regarding memory it is of great interest to know what cellular mechanisms are engaged in astrocytes and how that affects neuronal signaling.

Kim and colleagues addressed this important question in several ways. First, they tested if glutamatergic synaptic transmission and its plasticity in the CA1 region of the hippocampus are affected after prolonged optogenetic stimulation of astrocytes. They indeed identified deficits of long-term potentiation and depression (LTP and LTD) of synaptic transmission, which are thought to be basic synaptic mechanisms that underly learning. Because LTP and LTD are typically triggered by activity of glutamate receptors of the N-methyl-D-aspartate (NMDA) subtype at many synapses [7], the authors analyzed NMDA receptor activity next. They found reduced activity of NMDA receptors after stimulation of synaptic glutamate release and a reduction of NMDA receptors at the cell surface. Collectively, these experiments reveal a chain of events triggered by repeated and prolonged optogenetic stimulation of astrocytes that ultimately reduce learning by impairing the cellular machinery supporting learning. These findings further emphasize that astrocytes can profoundly, and at many levels, modify synaptic transmission and its plasticity with behavioral consequences (for review [1–3]).

Digging deeper, the authors also discovered that both stimulation paradigms increase the expression of the proinflammatory cytokines IL-1beta and TNF-alpha and of lipocalin-2 (LCN2). Stimulation also activated microglia and induced a reactive phenotype of astrocytes, which was assessed by investigating GFAP expression and astrocyte morphology. Appearance of reactive astrocytes, or astrocyte reactivity, is typically associated with brain diseases, injury or infection and describes the remodeling of astrocytes regarding their morphology, molecular biology, and function [8]. Induction of astrocyte reactivity by optogenetic and chemogenetic stimulation is an unexpected finding and highlights the necessity to test if such experimental manipulations lead to the induction of pathophysiological conditions. It is noteworthy that in the present study, this was not caused by the viral expression of chemogenetic and optogenetic tools but by the stimulation of the astrocytes itself. Regarding the mechanisms leading to astrocyte reactivity, the authors focus on LCN2, which fulfills many functions throughout the body including the brain, where it is implicated in neuroinflammation and neurodegeneration and a potential therapeutic target for these conditions [9]. It is therefore particularly interesting that in the study by Kim and colleagues, administration of LCN2 can reproduce important effects of repeated and prolonged optogenetic astrocyte stimulation, whereas LCN2 deficiency is overall protective.

**Fig 1:**
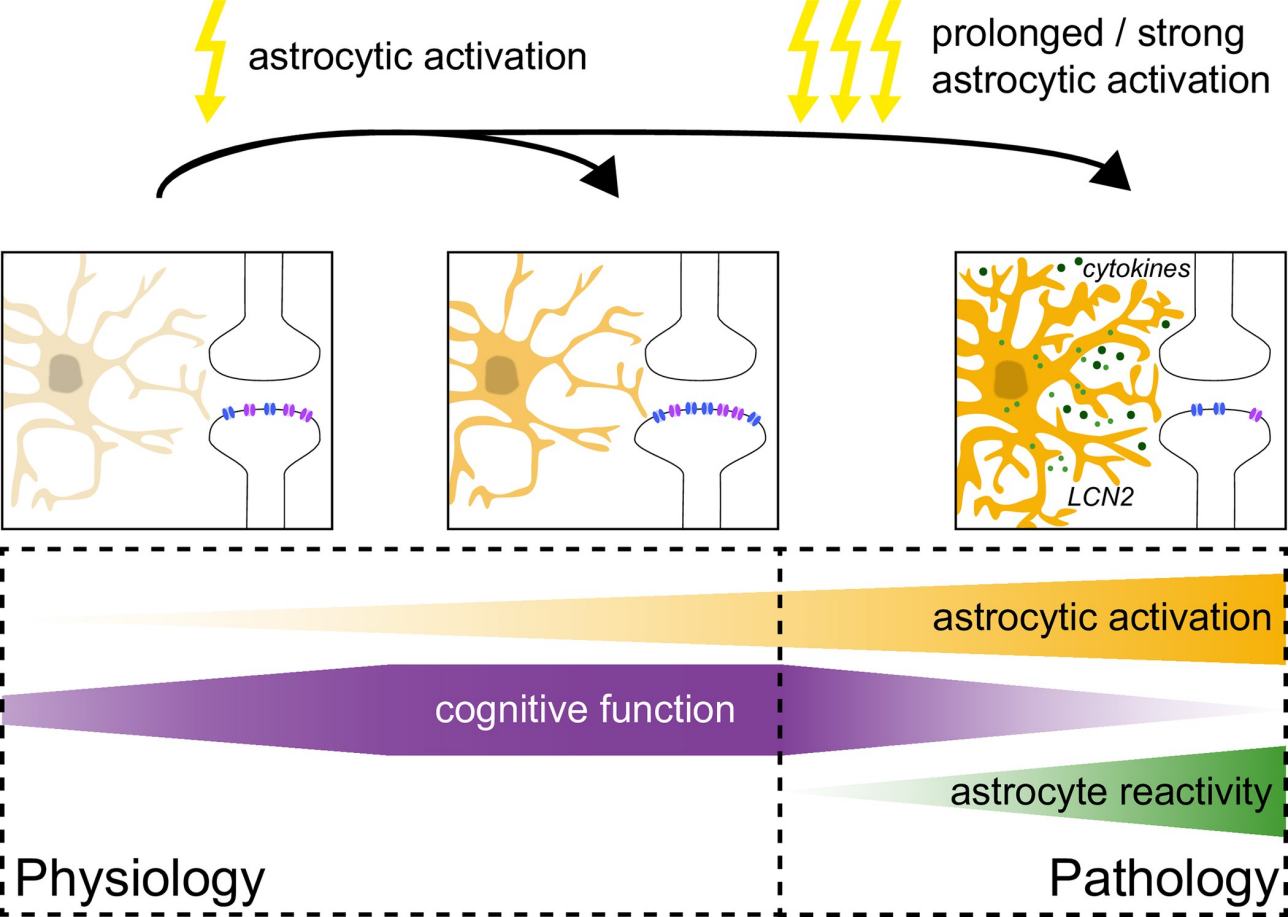
Activity-dependent modulation of cognitive function by astrocytes. Under physiological conditions (left side), the activity of astrocytes plays an important role in modulating synaptic transmission and its plasticity and ensures adequate memory encoding and retrieval. In line with that, experimental manipulations of astrocyte activity can modify many aspects of cognition (see main text). However, if astrocyte activation is prolonged and/or strong enough (right side) [6], this can induce a pathological, so-called reactive phenotype of astrocytes involving cytokine and LCN2 signaling. This causes an impairment of synaptic plasticity and of cognitive functions in mice.

Thus, the emerging picture is that repeated and prolonged stimulation of astrocytes leads to a reactive astrocyte phenotype in an LCN2-dependent manner, which leads to reduced synaptic plasticity and a cognitive impairment in mice (**Fig 1**). There are many interesting follow-up questions to the study by Kim and colleagues and opportunities for further important research. For example, it is increasingly believed that many aspects of astrocyte reactivity are specific to the diseases in which they occur, or specific to the initial trigger, even though there are clearly features that are common across diseases [8]. An interesting question is therefore, which disease or group of diseases leads to an astrocyte reactivity that is close or identical to the reactivity induced by Kim and colleagues and therefore shares the pathogenic mechanism? Or the other way around, which brain diseases or conditions with astrocyte reactivity would benefit most from the manipulation of LCN2 signaling [9]? Another example is related to the specialization of astrocytes across and within brain regions (for review [10]): A now open question is to what extent the astrocyte reactivity and its impact on synapse function shown by Kim and colleagues is varying between brain regions and synaptic circuits and whether such heterogeneity could provide insights into when and why optogenetic and chemogenetic astrocyte stimulation is of physiological or pathophysiological significance.
